# Increased Risk of Systemic Lupus Erythematosus in Patients With *Helicobacter pylori* Infection: A Nationwide Population-Based Cohort Study

**DOI:** 10.3389/fmed.2019.00330

**Published:** 2020-01-29

**Authors:** Meng-Che Wu, Pui-Ying Leong, Jeng-Yuan Chiou, Huang-Hsi Chen, Jing-Yang Huang, James Cheng-Chung Wei

**Affiliations:** ^1^Institute of Medicine, Chung Shan Medical University, Taichung, Taiwan; ^2^Division of Gastroenterology, Children's Medical Center, Taichung Veterans General Hospital, Taichung, Taiwan; ^3^Division of Allergy, Immunology and Rheumatology, Chung Shan Medical University Hospital, Taichung, Taiwan; ^4^School of Health Policy and Management, Chung Shan Medical University, Taichung, Taiwan; ^5^Department of Medical Research, Chung Shan Medical University Hospital, Taichung, Taiwan; ^6^Graduate Institute of Integrated Medicine, China Medical University, Taichung, Taiwan

**Keywords:** *Helicobacter pylori*, systemic lupus erythematosus, cohort study, population based, longitudinal health insurance research database

## Abstract

*Helicobacter pylori* (HP) infection is associated with systemic lupus erythematosus (SLE), but the related results have been controversial. Therefore, this study investigated the association between HP infection and SLE by using a nationwide longitudinal population-based cohort. We identified 41,651 patients with HP infection and 83,302 matched controls between 2000 and 2013 from the Longitudinal Health Insurance Research Database of the National Taiwan Insurance Research Database. Age, gender, comorbidities, and medical visits were matched at a 1:2 ratio by using propensity score analysis. The adjusted hazard ratio (aHR) of SLE was calculated by multiple Cox regression. Furthermore, sensitivity test and stratified analysis were performed. The SLE incidence rate was 1.17 [95% confidence interval (CI): 0.89–1.54] per 100,000 person-months in the HP cohort, and the hazard ratio was 1.63 (95% CI: 1.12–2.37) in comparison with the propensity score-matched control cohort. After multivariate adjustment, patients with HP infection had a significantly high overall aHR (1.58; 95% CI: 1.08–2.30) of SLE. Stratified analysis revealed the aHR of 8.23 (95% CI: 1.77–38.32) in patients <30 years old, and the *p* for interaction between age and HP infection was 0.039. For age–sex subgroup analysis, the highest aHR was 12.74 (95% CI: 1.55–104.59) in young (aged <30 years) female patients with HP infection. HP infection is associated with a 1.63-fold increased SLE risk, particularly with female patients aged <30 years. Future research is required to elucidate the underlying mechanism of this association.

## Introduction

Systemic lupus erythematosus (SLE) is a chronic autoimmune disease of unclear etiology which can affect the skin, serous membranes, joints, heart, lungs, kidneys, nervous system, and other organs of the body. Several genetic and environmental factors might play a role in SLE pathogenesis (1, 2). Among the known environmental factors, infections play a pivotal role in triggering the development of autoimmunity (3–6). Exposure to certain environmental agents including bacteria, viruses, and protozoa in genetically susceptible people may act as the catalysts that initiate SLE. Of the infectious pathogens proposed as agents inducing autoimmunity, *Helicobacter pylori* (HP) is one of the most extensively investigated (7–9).

HP is the Gram-negative, spiral-shaped, and microaerophilic bacterium with flagella which colonizes human mucosa of the stomach. It causes one of the most common bacterial infections in humans. The infection of HP usually occurs during early childhood and lasts for a lifetime if left untreated (10, 11). Since its discovery in 1982, HP infection has been recognized as the main cause of chronic gastritis, peptic ulcer disease, stomach cancer, and mucosa-associated lymphoid tissue lymphoma, and it has been related with extragastric disorders including iron deficiency anemia, vitamin B12 deficiency, neurodegenerative disorders, and metabolic syndrome (12, 13). It may be associated with various autoimmune pathogeneses, such as Sjogren's syndrome, rheumatoid arthritis, primary immune thrombocytopenia, autoimmune gastric atrophy, and autoimmune thyroiditis. Conversely, evidence exists that it may prevent the development of autoimmune diseases, such as SLE, autoimmune gastritis, multiple sclerosis, and inflammatory bowel diseases (8, 14, 15).

The epidemiology relation between HP and SLE is disputed, and results reported by published studies are inconsistent. Previous investigations using mouse models have shown that HP urease exposure can lead to anti-ssDNA antibody production (16). However, another case–control study compared the HP seropositivity prevalence in 466 SLE patients with a matched control group and discovered that SLE patients were less likely to be seropositive (36.5%) for HP in comparison with the healthy controls (42.9%). Subgroup analysis showed that HP exposure may prevent the development of SLE in the African American female population (17). Whether HP-infected individuals could be prone to or protected against SLE is unknown. Thus, whether HP is a friend or foe needs further research. Moreover, real-world population-based epidemiological studies are lacking. Hence, we investigated the association between HP infection and SLE through a retrospective cohort study at a nationwide level in this study.

## Methods

### Study Design and Population

**Figure 1:**
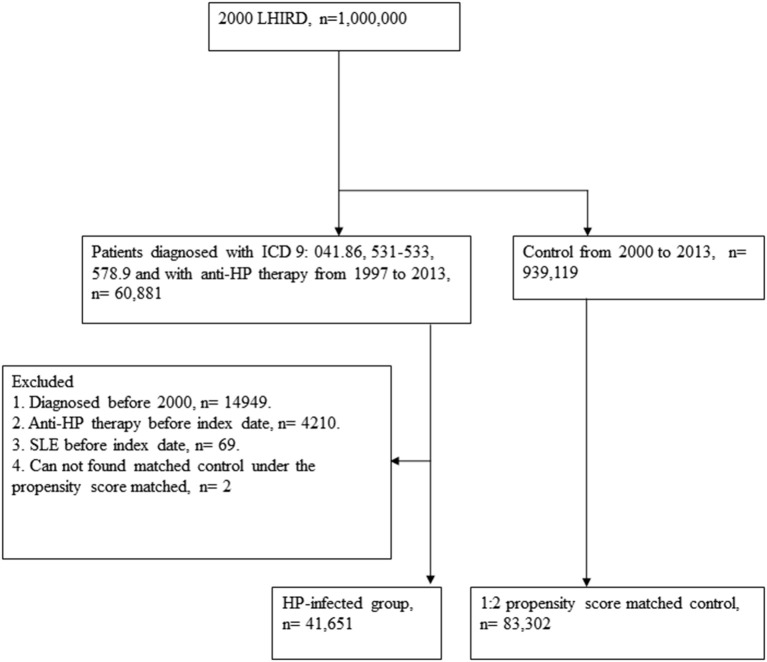
Study flowchart.

A retrospective cohort study was designed to analyze the association between HP infection and SLE. The flowchart is depicted in Figure 1. We accessed the Longitudinal Health Insurance Research Database (LHIRD) with one million randomly sampled individuals from the National Taiwan Insurance Research Database (NHIRD), a nationwide population-based insurance system, which enrolled 99% of the Taiwanese population and stored the medical claim records between 1997 and 2013 (18, 19). Moreover, LHIRD is one of the largest databases of the administrative medical care system (20). The incidence, prevalence, and correlations of selected factors can be determined by using this database. Diagnoses of patients are recorded according to the *International Classification of Diseases*, Ninth Revision, Clinical Modification (ICD-9-CM). Moreover, the demographic data, inpatient and outpatient expenditure claims, and other clinical information of patients are also recorded in this database. To prevent the confounding bias that often exists in the observational study design, we performed propensity score matching of selected variables to control the deviations. Thousands of studies have been published on the basis of this database (19). The study was conducted with the approval of the Institutional Review Board (IRB) of Chung Shan Medical University Hospital (IRB number CS15134).

### Exposure Definition of HP Infection and Controls

We identified patients diagnosed with HP infection (ICD-9-CM: 041.86), peptic ulcer (ICD-9-CM: 531–533), or hemorrhage of the gastrointestinal tract (ICD-9-CM: 578.9) who received anti-HP therapy from 1997 to 2013. According to the National Health Insurance system, the treatments of HP were confirmed by upper endoscopy with biopsy-based tests. Anti-HP therapy with triple or quadruple therapy contains proton pump inhibitor or H2 receptor blocker, plus amoxicillin or tetracycline, plus clarithromycin or metronidazole, with or without bismuth salts. The combination of drugs was prescribed in the same order for 7–14 days (details of all eligible HP eradication regimens were reported previously) (21). The first date of HP infection (ICD-9-CM: 041.86), peptic ulcer (ICD-9-CM: 531–533), or hemorrhage of the gastrointestinal tract (ICD-9-CM: 578.9) diagnosis was defined as the index date. Patients were excluded if they were ever diagnosed as having SLE before the index date, were diagnosed before 2000, underwent anti-HP therapy before the index date, or could not find a matched control under the propensity score.

For the control group, individuals registered in the LHIRD who did not receive anti-HP therapy were selected as candidates. To resolve the possible effect of confounding bias of comorbidities on incidence of SLE, control participants were 2:1 propensity score-matched with HP-infected patients by an 8-to-1 digit greedy matching algorithm. The index date for the controls was according to the respective matched cases. In this procedure, the probability was estimated by logistic regression model with the predictors, including by age, gender, urbanization level, income level, length of hospital stay (within 2 years before the index date), and comorbidities that are listed in Table 1. Finally, 41,651 patients with anti-HP therapy and 83,302 propensity score-matched controls were considered for the analysis.

**Table 1:** Baseline characteristics of study groups after propensity score matching.

	**Control (*n* = 83,302)**	**HP-infected group (*n* = 41,651)**	**Standardized difference**
Sex			0.01812
Female	37,632 (45.18%)	18,875 (45.32%)	
Male	45,670 (54.82%)	22,776 (54.68%)	
Age			0.01883
<30	8,832 (10.60%)	4,651 (11.17%)	
30–45	24,299 (29.17%)	12,151 (29.17%)	
45–65	37,272 (44.74%)	18,501 (44.42%)	
≥65	12,899 (15.48%)	6,348 (15.24%)	
Urbanization			0.00577
Urban	50,941 (61.15%)	25,555 (61.36%)	
Suburban	24,336 (29.21%)	12,149 (29.17%)	
Rural	8,025 (9.63%)	3,947 (9.48%)	
Low income	320 (0.38%)	179 (0.43%)	0.00717
Length of hospital stays^‡^			0.00779
0 day	70,333 (84.43%)	35,197 (84.5%)	
1–6 days	7,980 (9.58%)	4,031 (9.68%)	
≥7 days	4,989 (5.99%)	2,423 (5.82%)	
Comorbidity^‡^			
Rheumatoid arthritis	939 (1.13%)	509 (1.22%)	0.00880
Sjogren's syndrome	472 (0.57%)	252 (0.61%)	0.00503
Hypertension	18,500 (22.21%)	9,148 (21.96%)	−0.00590
Diabetes mellitus	9,573 (11.49%)	4,681 (11.24%)	−0.00798
Hyperlipidemia	13,243 (15.90%)	6,484 (15.57%)	−0.00907
Coronary artery disease	8,307 (9.97%)	4,206 (10.1%)	0.00420
Osteoporosis	4,333 (5.2%)	2,206 (5.3%)	0.00425
Cerebral vascular accident	3,964 (4.76%)	1,988 (4.77%)	0.00068
Asthma	4,394 (5.27%)	2,263 (5.43%)	0.00704
Chronic obstructive pulmonary disease	9,465 (11.36%)	4,725 (11.34%)	−0.00057
Chronic kidney disease	867 (1.04%)	463 (1.11%)	0.00686
Chronic liver diseases	13,832 (16.6%)	6,781 (16.28%)	−0.00874
Chronic urticaria	5,204 (6.25%)	2,627 (6.31%)	0.00247
Tuberculosis	770 (0.92%)	421 (1.01%)	0.00883
Pneumonia	2,331 (2.8%)	1,192 (2.86%)	0.00384
Sepsis	419 (0.50%)	238 (0.57%)	0.00936
Herpes zoster	1,241 (1.49%)	640 (1.54%)	0.00384

‡ *The length of hospital stays and comorbidity were identified within 2 years before index date*.

### Outcome and Comorbidities

The SLE patients were identified by using the ICD-9-CM code of 710.0. The diagnostic accuracy of SLE was confirmed by including patients with the aforementioned diagnostic code having at least one hospital admission or three outpatient visits and using hydroxychloroquine (HCQ) within 1 year after diagnosis to fulfill the case definition. We considered the baseline comorbidities that were associated with the risk of developing SLE. Potential risk factors in comorbidity were listed in “Rheumatology 2002; 41: 563–571.” Therefore, we selected comorbidities including rheumatoid arthritis (ICD-9-CM: 714.0), Sjogren's syndrome (ICD-9-CM: 710.2), hypertension (ICD-9-CM: 401–405), diabetes mellitus (ICD-9-CM: 250), hyperlipidemia (ICD-9-CM: 272), coronary artery disease (ICD-9-CM: 410–414), osteoporosis (ICD-9-CM: 733), cerebral vascular accident (ICD-9-CM: 430–438), asthma (ICD-9-CM: 493), chronic obstructive pulmonary disease (COPD) (ICD-9-CM: 490–492 and 493–496), chronic kidney disease (ICD-9-CM: 585), chronic liver diseases (ICD-9-CM: 571 and 573), chronic urticaria (ICD-9-CM: 708.8 and 708.9), tuberculosis (TB) (ICD-9-CM: 011–018 and 1370), pneumonia (ICD-9-CM: 480–486), sepsis (ICD-9-CM: 038), and herpes zoster (ICD-9-CM: 053). Comorbidities were defined from the diagnostic code as having at least one hospital admission or two outpatient visits of a given disease within 2 years before the index date. These comorbidities were considered covariates in the multivariate analysis.

### Statistical Analysis

We used the chi-square test to analyze the demographic difference between the HP and control groups, and the hazard ratios (HRs) were calculated by the univariate and multiple Cox proportional hazard regression models. We considered a *p* < 0.05 as statistically significant. For evaluating the measurement precision, 95% confidence interval (CI) was used. The cumulative incidence probability curves of SLE were generated with the Kaplan–Meier method, and the log-rank test was applied to examine the difference between curves. The landmark analysis was conducted to observe the SLE risk in 0–12, 13–36, and ≥36 months from the index date. The age subgroup and sex subgroup analyses evaluated the potential interaction effect between age, sex, and HP infection on SLE risk. All data were processed by SAS (version 9.4; SAS Institute, Cary, NC, USA).

## Results

**Figure 2:**
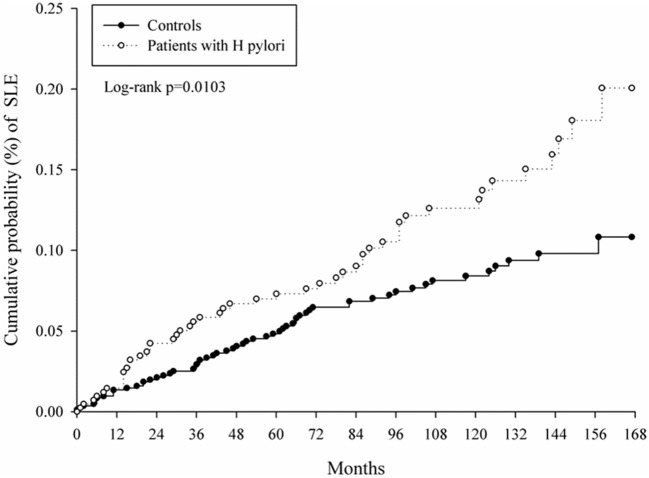
Kaplan–Meier curves of cumulative probability of systemic lupus erythematosus (SLE) in study groups.

At baseline, the frequencies of selected factors, including age, sex, monthly income, urbanization, and comorbidities, were averaged equally in each cohort (Table 1). No significant differences were observed in age, sex, and comorbidities. The mean follow-up periods for the HP cohort and control groups were 111 and 108 months, respectively. The incidence rate of SLE was significantly higher in the HP group than in the control group (1.17 vs. 0.72 per 100,000 person-months, crude relative risk: 1.63, 95% CI: 1.12–2.37, Table 2). After multivariate adjustment, a significant increase was observed in SLE risk in patients with HP infection [adjusted HR (aHR): 1.58, 95% CI: 1.08–2.30, Table 3]. Furthermore, stratified analysis (Table 4) revealed that the risk was higher in female patients (aHR: 1.7;95% CI: 1.13–2.57) than in male patients (aHR: 1.10; 95% CI: 0.43–2.83). On landmark analysis, the aHR of SLE for the HP-infected group was 2.38 (95% CI: 1.14–4.93) within 12–36 months of follow-up; nevertheless, the *p* for interaction between sex and follow-up time with HP infection was not statistically significant. Among the different age groups, patients with HP infection aged <30 years were at a significantly greater risk of developing SLE (aHR: 8.23; 95% CI: 1.77–38.32; *p* for interaction = 0.039; Table 4). By age–sex subgroup analysis (Table 5), the highest aHR (12.74; 95% CI: 1.55–104.59) was observed in female patients <30 years old with HP infection, and a decreasing trend of SLE risk was observed in patients aged >65 years with HP infection. Kaplan–Meier curves of the cumulative SLE rate are shown in Figure 2.

**Table 2:** Systemic lupus erythematosus (SLE) incidence in patients with *Helicobacter pylori* (HP) infection and controls.

	**Control (*n* = 83,302)**	**HP-infected group (*n* = 41,651)**
Follow-up person-months	8,323,634	4,275,515
New SLE case	60	50
Incidence rate^*^ (95% CI)	0.72 (0.56–0.93)	1.17 (0.89–1.54)
Crude relative risk (95% CI)	Reference	1.63 (1.12–2.37)

* *Incidence rate per 100,000 person-months*.

**Table 3:** Multiple Cox proportional hazard regression for the estimation of adjusted hazard ratios (aHRs) on systemic lupus erythematosus (SLE).

**Variable**	**aHR (95% CI)**
HP-infected group (ref: control)	1.579 (1.084–2.299)
Sex (ref: female)	
Male	0.199 (0.121–0.329)
Age (ref: 30–45)	
<30	1.021 (0.510–2.042)
45–65	1.026 (0.640–1.645)
≥65	0.870 (0.429–1.765)
Urbanization (ref: urban)	
Suburban	1.030 (0.675–1.570)
Rural	0.922 (0.472–1.799)
Length of hospital stays (ref: 0 day)	
1–6 days	1.090 (0.593–2.005)
≥7 days	2.203 (1.197–4.055)
Comorbidity	
Rheumatoid arthritis	5.422 (2.809–10.466)
Sjogren's syndrome	5.536 (2.342–13.083)
Hypertension	1.161 (0.687–1.961)
Diabetes mellitus	0.915 (0.492–1.704)
Hyperlipidemia	0.874 (0.500–1.528)
Coronary artery disease	0.643 (0.309–1.337)
Osteoporosis	1.160 (0.637–2.112)
Cerebral vascular accident	1.119 (0.471–2.661)
Asthma	0.980 (0.442–2.174)
Chronic obstructive pulmonary disease	1.082 (0.609–1.923)
Chronic kidney disease	-
Chronic liver diseases	2.188 (1.419–3.374)
Chronic urticaria	1.703 (0.951–3.051)
Tuberculosis	0.893 (0.121–6.606)
Pneumonia	0.527 (0.125–2.215)
Herpes zoster	2.780 (1.124–6.873)

**Table 4:** Sensitivity analysis for the adjusted hazard ratios (aHRs) stratified by follow-up time, sex, and age groups.

	**Incidence rate (95% CI) of SLE**		
**Subgroups**	**Control**	**HP-infected group**	**aHR (95% CI)**
**FOLLOW-UP TIME**			
0–12 months	0.13 (0.07–0.24)	0.14 (0.06–0.31)	1.053 (0.389–2.853)
12–36 months	0.16 (0.09–0.27)	0.37 (0.23–0.61)	2.370 (1.138–4.937)
36 months	0.49 (0.36–0.67)	0.69 (0.48–1.00)	1.357 (0.841–2.189)
*p* for interaction			0.3542
**SEX SUBGROUPS**			
Male	0.27 (0.15–0.48)	0.31 (0.15–0.64)	1.097 (0.429–2.805)
Female	1.24 (0.93–1.64)	2.16 (1.60–2.92)	1.700 (1.126–2.567)
*p* for interaction			0.4372
**AGE AT INDEX DATE**			
<30 years old	0.21 (0.05–0.82)	1.75 (0.91–3.36)	8.232 (1.769–38.316)
30–44 years old	0.58 (0.35–0.96)	1.14 (0.69–1.89)	1.812 (0.882–3.724)
45–64 years old	0.85 (0.60–1.21)	1.24 (0.83–1.87)	1.436 (0.836–2.464)
≥65 years old	1.07 (0.61–1.89)	0.50 (0.16–1.57)	0.465 (0.131–1.656)
*p* for interaction			0.0386

**Table 5:** Systemic lupus erythematosus (SLE) risk in patients with *Helicobacter pylori* infection stratified by age–sex subgroups.

		**Incidence rate (95% CI) of SLE**		
**Age (years old)**	**Sex**	**Control**	**HP-infected group**	**aHR (95% CI)**
<30	Female	0.22 (0.03–1.57)	2.90 (1.38–6.09)	12.74 (1.552–104.588)
<30	Male	0.19 (0.03–1.37)	0.73 (0.18–2.92)	3.552 (0.321–39.305)
30–44	Female	1.23 (0.73–2.08)	2.60 (1.57–4.31)	1.954 (0.939–4.066)
30–44	Male	0.07 (0.01–0.49)	No SLE case	-
45–64	Female	1.41 (0.95–2.09)	2.12 (1.35–3.33)	1.482 (0.815–2.696)
45–64	Male	0.32 (0.14–0.71)	0.42 (0.16–1.12)	1.293 (0.364–4.595)
≥65	Female	1.53 (0.77–3.06)	0.73 (0.18–2.93)	0.465 (0.097–2.221)
≥65	Male	0.67 (0.25–1.79)	0.31 (0.04–2.21)	0.420 (0.039–4.513)

## Discussion

In the current study, we found a significantly high incidental SLE risk in patients with HP infection. In addition, the SLE incidence rate was higher in female patients <30 years old with HP infection. To the best of our knowledge, this is the first and largest epidemiological study to use a nationwide longitudinal population-based dataset to identify an increased SLE risk among patients with HP infection. Highlighting the association can improve early SLE diagnosis among patients with HP infection, particularly in female patients <30 years old. The epidemiologic interpretation for this relationship may provide insights into SLE pathogenesis. Furthermore, before treatment with steroid or non-steroidal anti-inflammatory drugs (NSAIDs), testing for HP may be considered in patients with SLE and particularly in patients expected to be taking steroid continuously.

HP induces autoimmunity by various mechanisms, such as molecular mimicry, polyclonal activation, epitope spreading, bystander activation, and superantigen (5). Amedei et al. demonstrated that HP antigens can stimulate cross-reactive T cells and induce autoimmune gastritis by molecularly mimicking H+,K+-adenosine triphosphatase (22). Yamanishi et al. have demonstrated B-1-cell activation using HP urease, leading to the creation of anti-ssDNA antibodies (16). In a recent animal study, Surawut et al. found that HP infection increased anti-dsDNA and lupus severity in the symptomatic FcγRIIb-deficient lupus mouse model (23). Few clinical studies have been conducted to understand the correlation between HP infection and SLE. In the Japanese population, Showji et al. reported that the titer of anti-HP antibody was lower in SLE than in other connective tissue diseases (24). Kalabay et al. investigated the prevalence of anti-HP antibodies in a variety of autoimmune rheumatic diseases and revealed HP prevalence to be comparable in patients with SLE and healthy controls (25). Moreover, Sawalha et al. investigated the seroprevalence of HP in 466 patients with SLE and the matched controls. They showed that the positive rate of HP in SLE patients was lower (36.5%) than that in healthy controls (42.9%). This negative correlation is even more pronounced in African American women with SLE compared with controls (38.1 vs. 60.2%; *p* = 0.0009). HP-seropositive African American women are more likely to develop SLE at older ages as compared with HP-negative patients with SLE. The mean ages of SLE in the seropositive and seronegative groups were 34.4 and 28 years, respectively. The authors believed that HP infection may have a protective effect on SLE in this specific population (17). However, this is a typical case–control design that does not clarify causality. Furthermore, the study found a significant correlation only between African and American women and showed that the correlation may be affected by age. Additionally, our study found that patients aged >65 years with HP infection had a lower risk of SLE but did not reach statistical significance. By contrast, one recent study by Prado et al. showed high HP frequency in patients with SLE through endoscopic and histopathological findings (26). These investigations have some drawbacks, but most of them have shown a negative correlation between HP infection and SLE. This difference may be due to the diversity in seroprevalence of HP infections in different countries of the world. Most of the studies have been performed with small study populations, mainly with HP serological testing and the case–control method, rather than through the cohort approach.

The immunopathological process by which HP increases the risk of developing SLE remains uncertain. Buzzelli et al. found that ulceration of gastric tissue induced by HP is associated with the increased alarming molecules, such as IL-33, which may act as a classic cytokine or transcription factor (27). This might modify the immune system to restore homeostasis of epithelial cells. In addition, IL-33 has a potential role in autoimmune disease development (28). Chmiela et al. reviewed that bioinformatic analysis shows homology between the amino acid sequences of HP CagA and human Hsp60, and IL-33. It is hypothesized that host Hsp60 and IL-33 can be both targeted by antibodies stimulated by HP CagA-positive strain infection, which may affect the gastric inflammatory response (29). They also hypothesized that the long-term exposure of specific memory cells to these HP compounds would allow for continued induction and transformation to effector lymphocytes that may be associated with autoimmune-mediated tissue destruction (29). Recently, Mu et al. reviewed that leaky gut may be a dangerous signal for autoimmune disease (30). HP has been shown to directly enhance epithelial permeability by redistributing the tight junction protein ZO-1. In theory, HP infection could play an important role in the pathogenesis of SLE by triggering alterations in gastric and/or intestinal permeability or by inducing immunological derangements to cause autoimmunity. Also, recent studies reported that pathogenic bacteria can destroy intestinal barrier and trigger SLE (31–33). Moreover, our findings supported the association between HP infection and increased SLE risk. We speculated that the increased incidence of SLE among patients with HP infection could be due to chronic inflammation and immune dysregulation through HP infection.

The strengths of this research are its large sample size and use of a nationwide longitudinal population-based database. Therefore, our study is free from selection and recall bias, which made examining our hypothesis feasible. Nevertheless, a number of limitations must be noted. First, the NHIRD does not contain patients' socioeconomic status and personal health behaviors such as smoking habits, alcohol use, coffee consumption, family history, and inflammatory biomarkers. The association between HP infection and SLE could be biased by these confounding factors. Although we adjusted for various comorbidities and performed propensity score matching, these unmeasured confounders might affect our results. Second, the NHIRD database only recorded patients receiving HP therapy; therefore, the severity and duration of HP infection could not be evaluated in this investigation. Data on the virulence factors of HP strains were unavailable. With symptoms that are difficult to notice, the HP infection prevalence could be underestimated. It is possible that we could have enrolled asymptomatic HP infection patients in the control group. However, this bias would result in an underestimation of the SLE risk in patients with HP infection and would not alter our results. Third, with the variation in clinical manifestations and diagnosis, SLE diagnosis may be disregarded by clinicians. The SLE event was defined as diagnosis with the ICD-9-CM code of 710.0 (at least one hospital admission or three outpatient visits) and using HCQ within 1 year after diagnosis. As a result, the data for the diagnosis of SLE should be accurate. However, the number of new SLE cases was rather small despite the large number in both cohorts. In addition, this inclusion criterion did not involve some SLE patients who might be intolerant to HCQ and might reduce the number of patients with SLE, thus reducing the power in statistical analysis. On the other hand, there were still lupus-like cases misclassified into the SLE event in our study. The risk ratio might be biased when the misclassification was differential on the definition of SLE event in this study. Fourth, whether anti-HP therapy can reduce SLE risk is unable to be clarified by this study. According to our observations about general practice patterns in Taiwan, patients with HP infection almost always receive anti-HP therapy; therefore, patients with HP infection without anti-HP therapy are quite few. Further studies about whether the disease activity of SLE is related to HP infection and whether the eradication of HP in SLE patients helps maintain SLE remission should be carried out. Finally, it remains unclear whether the finding in our investigation can be extrapolated to other ethnic groups, since most patients were Taiwanese. This finding should be more reliable in the Asian population rather than other ethnic populations. More clinical investigations should be performed in patients from other countries in order to prove the association between HP and SLE.

## Conclusion

The 13-years population-based cohort proved a high SLE risk in patients having HP infection, particularly among female patients aged <30 years. Future research may elucidate the possible mechanisms for these associations. Clinicians should provide appropriate monitoring for SLE in patients with HP infection.

## Data Availability Statement

All datasets generated for this study are included in the manuscript.

## Ethics Statement

The studies involving human participants were reviewed and approved by the Institutional Review Board of Chung Shan Medical University Hospital (approval number CS15134). Written informed consent for participation was not required for this study in accordance with the national legislation and the institutional requirements.

## Author Contributions

M-CW drafted the manuscript. JW revised the manuscript critically. All authors approved the final version of the manuscript, contributed to the conception, design, and interpretation of the work substantially.

### Conflict of Interest

The authors declare that the research was conducted in the absence of any commercial or financial relationships that could be construed as a potential conflict of interest.
